# The Study of Sarcoma Microenvironment Heterogeneity Associated With Prognosis Based on an Immunogenomic Landscape Analysis

**DOI:** 10.3389/fbioe.2020.01003

**Published:** 2020-08-21

**Authors:** Jin Deng, Weiming Zeng, Wei Kong, Yuhu Shi, Xiaoyang Mou

**Affiliations:** ^1^College of Information Engineering, Shanghai Maritime University, Shanghai, China; ^2^Department of Biochemistry, Rowan University and Guava Medicine, Glassboro, NJ, United States

**Keywords:** sarcoma microenvironment, heterogeneity, immunogenomics, pathway, prognosis

## Abstract

Microenvironment-driven tumor heterogeneity causes the limitation of immunotherapy of sarcomas. Nonetheless, systematical studies of various molecular levels can enhance the understanding of tumor microenvironment (TME) related to prognosis and provide novel insights of precision immunotherapy. Three prognostic-related TME phenotypes were identified by consensus clustering of the relative infiltration of 22 immune cells from 869 samples of sarcomas. Additionally, integrative immunogenomic analysis is applied to explore the characteristics of different TME groups. The results revealed that most of the immune cell infiltration is higher in the better prognostic group, which are more affected by lower DNA methylation levels and fewer copy number variations in the worse prognostic group. The signaling pathway crosstalk analysis suggested that the changes in the TME will cause considerable variation in the flow of information between pathways, especially when the degree of relative infiltration of immune cells is low, patient’s endocrine system may also be significantly affected. Also, the endogenous competitive network analysis indicated that several differentially expressed long non-coding RNAs (lncRNAs) associated with the prognosis or tumor recurrence of sarcoma patients affected the regulatory relationship between miRNAs and different genes when the sarcoma microenvironment changes. In summary, the significant relationship between genetic alterations and prognostic-related TME characteristics in sarcomas were determined in this study. These findings may provide new clues for the treatment of sarcomas.

## Introduction

Sarcoma refers specifically to malignant tumors caused by problems in human muscle and connective tissue, the characters of which are mainly rapid disease progression, short duration, and high-frequency metastasis (Daw et al., 2015; Giuliano et al., 2016; Steele and Pillay, 2019; Deng et al., 2020b). The molecular characteristics of sarcomas result in internal heterogeneity that influences personalized medicine (Farzana and Haass, 2018; Deng et al., 2020a). At the molecular level, the tumor heterogeneity is mainly regulated by the features of different subpopulations of cells with distinct genomic transcriptome, epigenome, and proteome alterations (Tirosh et al., 2016; Grzywa et al., 2017). Therefore, it is necessary to distinguish the tumor types of patients accurately and then identify critical biomarkers related to the survival rate.

Primary sarcomas contain varying numbers of tumor-infiltrating lymphocytes, and the abundance of macrophages and T cells is much lower than other malignant bone tumors, so it is not possible to perform a comprehensive quantitative characterization of the immune subpopulations in the tissue by standard immunohistochemistry (Berghuis et al., 2016; Paydas et al., 2016). The Cancer Genome Atlas (TCGA) Research Network has comprehensively integrated genomic characterization of adult soft tissue sarcomas, and previous studies have used some sarcoma gene expression data to infer the degree of infiltration (The Cancer Genome Atlas Research Network,, 2017; Huang et al., 2019; Stahl et al., 2019). However, these studies have only identified the associated characteristic genes after inferring the relative infiltration abundance of immune cells based on the level of single gene expression data, without identifying microenvironment phenotypes from the aspect of multiple dimensional data. As a result, it is impossible to explore the pathogenic mechanism between different microenvironment phenotypes in depth. Besides, due to the small sample size of most experiments, there may be some false positives in the inferred results, and it is neither universal nor representative. Moreover, an increasing body of literature suggests that genomic factors of cell-level and intercellular relationships in tumor microenvironment (TME) play a vital role in cancer progression and treatment response (Quail and Joyce, 2013; Kalluri, 2016; Mantovani et al., 2017; Zeng et al., 2019). Meanwhile, the whole landscape of sarcomas microenvironment phenotypes remains unknown, which results in a lack of awareness of the microenvironment of sarcomas. Thus, there is a tremendous need to comprehensively explore the TME phenotypes of sarcomas from a multi-dimensional perspective.

Furthermore, the single genes are sensitive to environmental or other factors, and genes often perform specific biological functions in the form of pathways (Khatri et al., 2012; Kong et al., 2014). Researchers have also proved the significance of tumor-related structures and upregulated signaling pathways in cancer cells and TMEs (Quail and Joyce, 2013; Kalluri, 2016). Compared to pathways, the more microscopic perspective is mutation performances on genes. Genetic profiling has revealed that the mutation frequency of Dedifferentiated Liposarcoma ranged across 0.1–3/Mb and Leiomyosarcoma ranged across 0.2–9/Mb (The Cancer Genome Atlas Research Network,, 2017). Some studies have analyzed the connection between mutation alterations and immune filtration to discover those alterations that influenced relative immune infiltration in several cancers (Rooney et al., 2015; Safonov et al., 2017; Thorsson et al., 2018; Li and Cai, 2019; Zeng et al., 2019). Incorporating link information of microRNA and gene in feature selection was usually applied to identify the potential tumor biomarkers (Yang et al., 2017, 2018; Liu and Yang, 2018). Meanwhile, the crosstalk between the tumor cells and tumor-infiltrating immune cells is generally modulated by the competing endogenous RNA (ceRNA) networks composed of microRNAs (miRNAs), messenger RNAs (mRNAs), and long non-coding RNAs (lncRNAs; Leonardo et al., 2011; Deng et al., 2018b; Huang et al., 2019). However, few studies conducted a differential genomic analysis of sarcomas among different TMEs, and the comprehensive landscape of cells infiltrating the TME of sarcomas has not yet been elucidated.

In this study, we extrapolated the relative infiltration signatures of 22 immune cells based on the gene expression profile of samples from multiple platforms and then extracted three TME subtypes associated with the prognosis of sarcoma patients. According to exhibiting the correlation of molecular data in various dimensional spaces, the characteristics of different TME were identified from signaling pathways, DNA methylation, copy number variation (CNV), and ceRNA networks related to differentially expressed genes (DEGs) among three TMEs.

## Materials and Methods

### Sarcoma Datasets and Preprocessing

We systematically collected 869 sarcoma gene expression datasets from the National Center for Biotechnology Information and TCGA. Specifically, multiple types of 263 sarcoma samples data under Illumina RNAseq technology and the corresponding clinical information were acquired on the TCGA website^1^. For the National Center for Biotechnology Information website, the Medical Subject Headings (MESH) search was applied to discover related datasets. The instruction of MESH is (((**survival** OR **prognosis** OR **prognostic** OR **outcome** OR **death** OR **relapse** OR **recurrence**))) AND ((**tissue cancer** [MeSH Terms]) OR ((((((**tissue cancer**[Title]) OR **tissue sarcoma**[Title]) OR **tissue neoplasm**[Title]) OR **tissue tumor**[Title]) OR **tissue carcinoma**[Title]) OR **sarcoma**[Title])]) AND (**Homo sapiens**). Under this instruction, 126 subjects were obtained, and then GSE75885 (Delespaul et al., 2017) and GSE71121 (Lesluyes et al., 2016) that contain more clinical information were selected. GSE71121 consists of GSE71118, GSE71119, and GSE71120. The patients related to these five items were applied to estimate fractions of TME cells, basic information of which was shown in Table 1.

**TABLE 1:** Basic characteristics of patients used for estimating fractions of TME cells.

ID	Platform	No. of patients	No. of samples	Gender		Age		No. of metastasis	No. of local recurrences
				
				Female	Male	Mean	95% CI		
GSE75885	Illumina HiSeq 2000 (Homo sapiens)	112	117	56	56	65	62–67	38/112	34/112
GSE71118	[HG-U133_Plus_2] Affymetrix Human Genome U133 Plus 2.0 Array	217	312	108	109	62	60–64	80/217	49/217
GSE71119	Illumina HiSeq 2000 (Homo sapiens)	95	136	41	54	65	63–67	44/95	32/95
GSE71120	Illumina HiSeq 2000 (Homo sapiens)	41	41	26	15	63	58–66	6/41	8/41
TCGA Sarcoma	Illumina RNAseq	261	263	142	119	61	59–63	99/261	53/261

In order to make the data from different platforms more similar and more comparable between samples, the fragments per kilobase million (FPKM) value of microarray data were converted to transcripts per million value.

### Inference of Infiltrating Cells in the TME

In order to quantify the proportion of immune cells in samples of sarcomas, CIBESORT algorithm and LM22 gene characteristics were applied to analyze the infiltration ratio of 22 human immune cell phenotypes, including B cells, T cells, natural killer cells, macrophages, etc. CIBERSORT is a tool for deconvolution of the expression matrix of immune cell subtypes based on the principle of linear support vector regression (Newman et al., 2015). At present, microarray and RNAseq data can be used to estimate the immune cell infiltration. Therefore, according to the uploaded gene expression data and the default 22 immune cell signatures, the infiltration of 22 immune cells in each sample was inferred.

### Consensus Clustering of Infiltrating Cells in TME

An unsupervised clustering method was used to classify all samples to represent different microenvironment groups after obtaining the relative infiltrating of immune cells in all samples. Consensus clustering is a commonly used research method for the study of cancer subtype classification, and this clustering method usually yields better results. The *ConsensusClusterPlus* R package (Stefano et al., 2003) is simple to operate and contains multiple methods, such as K-means, Pam, etc. Therefore, in this study, all samples were clustered into three clusters using consensus clustering with the K-means and Pam methods.

### DEGs Associated With TME Phenotypes

In order to identify DEGs between TME cell infiltration patterns, the limma package (Ritchie et al., 2015) that can analyze both microarray data and RNAseq data was used to obtain DEGs for samples under three types of TME, where the selected threshold was set to that significant *P* value is less than 0.01 and the adjusted *P* value is less than 0.05. We performed an intersection analysis of the three TME groups of DEGs using the Venn diagram to observe the similarities and differences of DEGs in types of TMEs.

### Signaling Pathway Crosstalk Analysis

Exploring the differences only considers a single gene that ignores the effects of mutual disturbances between genes. The dissimilar enriched pathways in three TMEs indicate that there will also exist some discrepancies in the crosstalk between the pathways. Signaling pathway impact analysis defines the contribution of each signaling pathway through two indicators of differential gene overexpression (*P*_*NDE*_) and abnormal disturbance (*P*_*PERT*_) in a given pathway, which were combined to define the global probability, *P*_*G*_ (Tarca et al., 2009; Deng et al., 2018a). The smaller the *P*_*G*_, the higher the significance of the pathway. All enriched pathways between every two groups were ranked based on the DEGs between types of TMEs, and then those significant signaling pathways with the global variable *P*_*G*_ less than 0.05 were extracted. Moreover, the distance correlation method was utilized to calculate the crosstalk values between high-contribution pathways that were recorded as *Rm*_1_ and *Rm*_2_, where *m_*i*_(i = 1,2)* represents different comparison groups, respectively, namely TME-A versus to TME-B, TME-A versus to TME-C, and TME-B versus to TME-C. Then, the distinction between *Rm*_1_ and *Rm*_2_ was calculated as the change of the crosstalk in each pair of comparison groups, that is, *R_*d*_ = Rm_1_–Rm_2_*, whose value ranges from –1 to 1. The absolute value of *R*_*d*_ describes the significance of the correlation between the two pathways, and the correlation gets stronger as the value becomes larger.

### Differential DNA Methylation Analysis

The corresponding methylation value, beta (β) represents the ratio of the methylated probe intensity to the total probe intensity. The DNA methylation data with a value of NA was removed, and then the rest of the methylation data of 265 sarcoma samples was divided into three groups based on the samples, including TME-A, TME-B, and TME-C. In order to be consistent with the analysis of gene expression data, the difference analysis of intergroup differential methylation probe data was also carried out using the *limma* R package. Those probes with the fold-change value greater than 1.25 and the false discovery rate values less than 0.01 were identified as hypermethylated, while those with fold-change values less than 0.8 and false discovery rate values less than 0.01 were identified as hypomethylated.

In order to explore the genomic region distribution of three TME groups, the performance on genomic region distribution was obtained by gene annotation classification and CpG annotation classification, including TSS1500, TSS200, 5’ untranslated region (UTR), 1st exon, Gene body, and 3’ UTR. After extracting all the significantly differential methylation sites, we classified all CpG sites according to region annotated information and calculated mean beta value for each classification in three TME groups. The differences between TME groups were compared using the two-sample *T* test. The number of common areas, the number of high methylations, and low methylations were also counted. Then, Fisher’s test and the chi-square test were applied to evaluate the remarkable difference between TME comparison groups in specific annotated regions.

### CNV Analysis

In addition to DNA methylation, genomic structural variation as one of epigenetic information is also crucial genetic information to be considered. The performance on copy number mutations in three microenvironment groups was explored in this study. CNV data of TCGA-sarcoma patients included samples, chromosomes, starting positions, ending positions of chromosomes, the number of probes, and segment mean. The CNV annotation file is downloaded from the UCSC Genome Browser: Annotation Database^2^. If the regions of probes contain the annotated regions, it is determined that the probe is located in the corresponding gene region. Next, the variation of the copy number of the region is determined according to the segment mean. Assuming that the value of the segment means is *x*, then the copy number is defined as *copynum = 2^(x + 1)*. According to the range of *copynum*, five cases are including: (1) If the value of *copynum* is greater than 3.5, the region is considered to have amplified two or more copy numbers; (2) If the value of *copynum* is greater than 2.5 and less than 3.5, it is considered to have amplified one copy number in the region; (3) If the value of *copynum* is greater than 1.5 and less than 2.5, it is considered that there is no change in the region; (4) If the value of *copynum* is between 0.5 and 1.5, it is considered to have been deleted one copy number in the region; and (5) If the value of *copynum* is less than 0.5, two copy numbers are considered to be deleted in the region. Finally, the chi-square test is used to evaluate the differences of CNVs among different TME groups as well as the expression value and frequency of annotated genes.

### Construction of ceRNA Network

The ceRNA network mainly explores the regulation and competition relationship of differential molecular composition in every two groups of TMEs. We first calculated significantly differentially expressed lncRNAs and miRNAs using the limma R package, and then used the mircode database (Jeggari et al., 2012) to find all the matching information for differentially expressed lncRNA, that is, differentially expressed miRNAs related to differentially expressed lncRNAs. In order to find the targeted genes related to differentially expressed miRNAs, the starbase database (Li et al., 2014) was applied to perform 3p and 5p annotation on miRNAs. For the labeled miRNAs, the corresponding regulatory genes were matched from three databases, including miRDB (Wong and Wang, 2015), miRTarBase (Chou et al., 2016) and TargetScan (Garcia et al., 2011). Finally, the ceRNA network between each pair of TME groups was constructed using the relationship between three types of differentially expressed RNAs.

## Results

### Identification of TME Subtypes of Sarcoma

**FIGURE 1:**
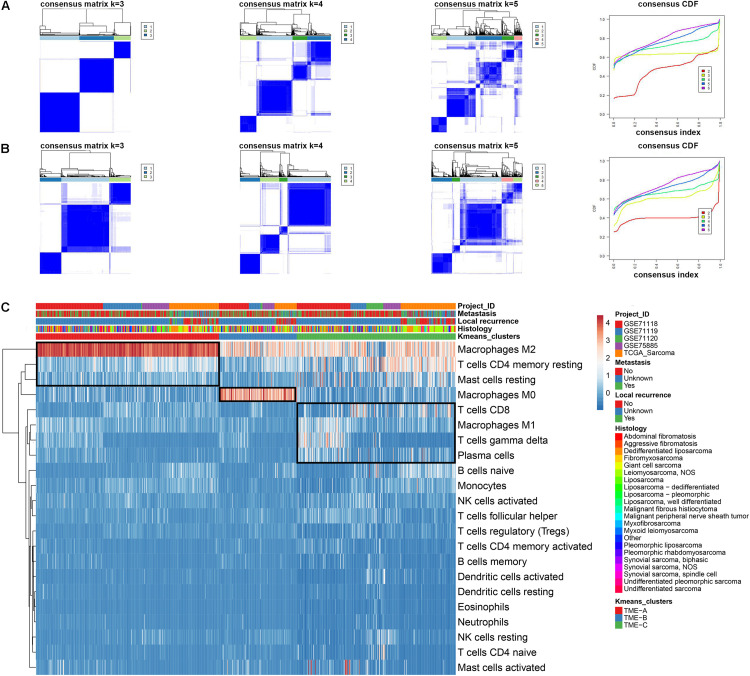
Consensus clustering of immune cell infiltration in 869 sarcoma samples. **(A)** Consensus clustering matrix for *k* = 3 to *k* = 5 using the K-means clustering method. The three heatmaps denote the consensus matrices of sample pairs. Each matrix item calculates the proportion of times that the pair of samples are clustered together across the resampling iteration. The consensus CDF curves are shown for different *k* from 2 to 6. **(B)** Consensus clustering matrix for *k* = 3 to *k* = 5 using pam clustering method. **(C)** The Infiltration performance of 22 immune cell types in three TME clusters and five independent sarcoma cohorts. Rows represent immune cells and columns represent samples. Euclidean distance and Ward linkage preform on the hierarchical clustering of all TME cells.

In this study, 869 samples from 5 datasets were used to evaluate the infiltration of 22 immune cells based on CIBERSORT to eliminate the biased effects of different platforms. The results of CIBERSORT was shown in Supplementary Table S1. In order to select the best clusters, hierarchical clustering was applied to perform unsupervised clustering analysis based on the immune cell infiltration information of all samples. The partitioning around medoid (pam) and K-means methods were also applied to analyze the stability of several cluster numbers, as shown in Figures 1A,B. The cumulative distribution function (CDF) curve is used to determine the *K* value when the cluster analysis result is most reliable. Generally, the *K* value with a small decline slope of the CDF curve is taken. Additionally, the heatmap plot was used to explore the distribution of samples between clusters and the distribution of immune cell infiltration, as shown in Figure 1C.

The heatmap obtained using the K-means clustering method with *k* = 3 is clearest from Figure 1. The slope of the CDF curve is also smaller than the others. Thus, the final cluster number is set to 3 according to the performance on the consensus clustering heatmap and CDF curve. These samples are divided into three TME groups, of which the first type includes 379 samples, the second type includes 161 samples, and the third type includes 329 samples. Also, the infiltration abundance of most immune cells did not exceed 1. Several patterns were also revealed among clustering results. Compared with other TME groups, the infiltration degree of Macrophages M2 was significantly higher in TME-A, while the infiltration abundance of T cells CD4 memory resting and Mast cells resting was significantly lower. Moreover, the infiltration degree of Macrophages M0 was higher in TME-B. The distribution of T cells CD8, Macrophages M1, T cells gamma delta, and Plasma cells in the samples was also significantly different in TME-B compared with other TME groups.

All the samples have been accurately classified into three subtypes of TME from the information from multiple data sources. The TCGA samples were divided into three groups, including 101 TME-A samples, 46 TME-B samples, and 109 TME-C samples. Considering that the TCGA-sarcomas data is more comprehensive and contains a variety of molecular data information of the sample from the same batch, the sarcoma data from the TCGA platform is independently analyzed for the transcriptome characteristics and clinical characteristics of microenvironment subtypes. The characteristics of pathological data, especially the subtypes of sarcomas, are also significantly different among TME subtypes. In this study, the Pearson chi-square test method was performed for clinical indicators and subgroups. The results showed that three types of factors, including metastasis, recurrence, and molecular type, were significantly associated with subgroups with *P* value of less than 0.001, which indicates that there are also differences in metastasis and local recurrence. It can also be clearly seen that the ratio of leiomyosarcoma regard as one of the subtypes of sarcomas is higher in TME-C.

### Characteristics of Three TME Groups

**FIGURE 2:**
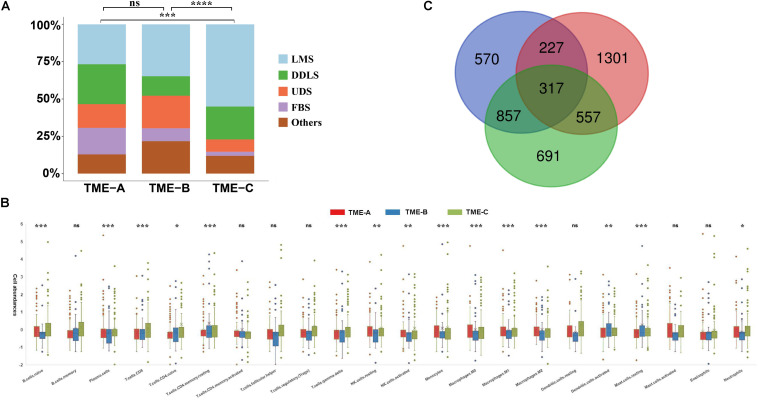
Characteristics of three TME groups. **(A)** The distribution of tumor histological types in three TME groups. The Fisher’s test was applied to verify the difference in the distribution of sarcoma histological types in TME groups. The *P* value is labeled with asterisks in each grid (****P < 0.0001, ***P < 0.001, **P < 0.01, *P < 0.05, and ns no significance). LMS: Leiomyosarcoma; DDLS: Dedifferentiated liposarcoma; UDS: Undifferentiated sarcoma; and FBS: Fibromyxosarcoma. **(B)** The relative abundances of 22 immune cell types in three TME groups. Z-score transformed the original cell abundances. Two vertical lines on each box represent Whisker upper and lower limits. The fork represents the mean and the horizontal line in the box represents the median. The points outside the box indicate the outlier points. The Kruskal–Wallis test is performed on the three TME groups for each immune cell type, where *P* value is labeled with asterisks above each box (***P < 0.001, **P < 0.01, *P < 0.05, and ns: no significance). **(C)** The Venn diagram of the overlapping DEGs between three groups.

The sarcomas contain over 10 histological subtypes in this study. In order to verify whether the sarcoma histological types will affect the overall results, the distribution of the samples in each TME group is presented in Figure 2A. Each group contains various histological types. The fisher’s test was also performed on the distribution of sarcoma histological types in TME groups. The results reveal the significant differences between TME-C and other groups. Especially, TME-C mainly includes two histological types, Leiomyosarcoma (55%), and Dedifferentiated liposarcoma (22%). Leiomyosarcoma also comprises a high proportion of both TME-A and TME-B.

Besides, the infiltration abundance of 15 immune cells is differential among the three groups, as shown in Figure 2B. The infiltration degree of Macrophages M2 in TME-A is significantly higher than that in the other two groups, while the infiltration abundance of T cells CD4 memory resting and Mast cells resting was significantly lower. The relative infiltration degree of Macrophages M0 is the highest in TME-B, while the relative infiltration degrees of Monocytes and Macrophages M1 are lowest among three TME groups. Moreover, T cells CD4 memory resting, B cell naïve, and Plasma cells showed higher relative infiltration in TME-C, and the distribution of infiltration of Mast cells resting, and T cells CD8 in the samples was also significantly different from TME-A and TME-B.

Furthermore, the Venn diagram was applied to calculate the acquisition of DEGs with the adjusted *P* value less than 0.05, and 317 DEGs were obtained in Figure 2C. The expression of most DEGs is a continuous decrease from TME-C to TME-A to TME-B.

### Survival Analysis of Three TME Groups

**FIGURE 3:**
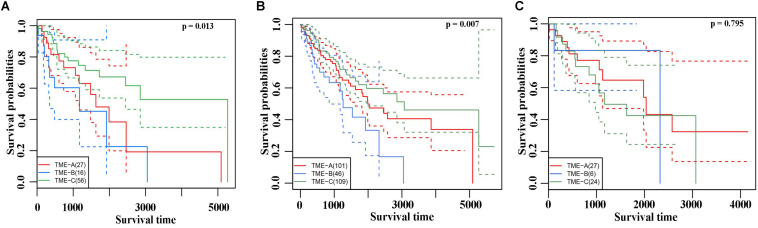
**(A)** The Kaplan–Meier curves of overall survival based on three TME groups. Different solid color denotes different group. The dashed line indicates a 95% confidence interval. **(B)** The Leiomyosarcoma samples in the three TME groups had significant differences in survival. **(C)** The Kaplan–Meier curves of overall survival based on Dedifferentiated liposarcoma samples.

The Kaplan–Meier curves of overall survival were performed on three TME groups in Figure 3A. Different solid color denotes different group. The dashed line indicates a 95% confidence interval. It revealed that there are also significant differences in the prognosis effects among three TME groups by calculating the survival rates of the three TME subtype samples, in which TME-C with better, TME-A with intermediate, and TME-B with worse prognoses.

In TME-C, more than half of the sarcoma histological types are Leiomyosarcoma. To verify that the difference in survival analysis was caused by sarcoma microenvironment rather than histological types, survival analysis for the Leiomyosarcoma in three TME groups was performed in Figure 3B. The result of overall survival analysis was the same as pan-sarcoma analysis, TME-C with better, TME-A with intermediate, and TME-B with worse prognoses. We have also conducted a survival analysis of Dedifferentiated liposarcoma samples in Figure 3C. However, there is no significant difference among the three TME groups. This may be due to the limitation of the small sample size of Dedifferentiated liposarcoma.

### Enrichment Performance and Pathway Crosstalk Analysis Among Three TME Groups

**FIGURE 4:**
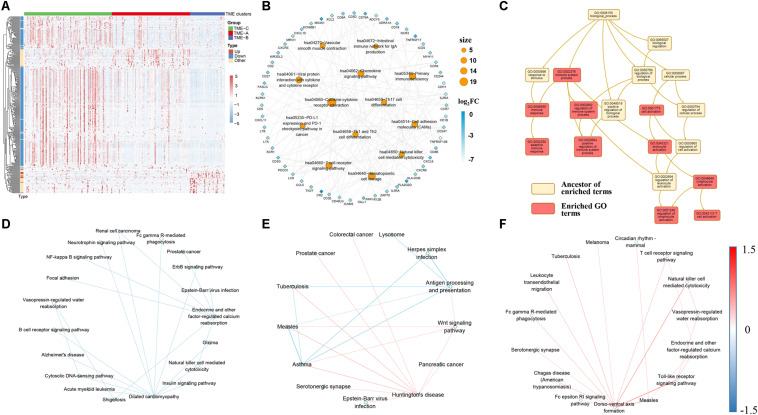
**(A)** The expression performance of DEGs in three TME groups. Log10 transformed the transcripts per million values of all DEGs. Pearson correlation used as a clustering distance method, and Euclidean distance was performed on the hierarchical clustering of all DEGs. Type represents the changes in expression of DEGs from TME-C to TME-A to TME-B. “Up” represents a continuous increase, “Down” represents a continuous decrease, and “Other” represents increase first then decrease or decrease first then increase. **(B)** The KEGG pathway enrichment network of DEGs. Orange circular nodes represent KEGG pathways. Diamond nodes represent DEGs. The size of the circle denotes the number of DEGs enriched in the pathway. The color of diamond notes denotes the degree of difference between TME-C and TME-B. **(C)** Enriched GO terms graph of DEGs and top-ranking neighbors. The yellow box represents the ancestor of enriched terms, and the red box represents enriched GO terms. **(D–F)** The crosstalk relationship of pathways in three TME groups, and the color of line represents the change of significant pathway correlation. **(D)** represents the crosstalk between TME-A and TME-B. **(E)** represents the crosstalk between TME-A and TME-C. **(F)** represents the crosstalk between TME-B and TME-C.

After obtaining 317 common significantly DEGs, the distribution of gene expression levels among TME groups was performed, as shown in Figure 4A. Kyoto Encyclopedia of Genes and Genomes (KEGG) enrichment analysis was performed on all genes using the *clusterProfiler* R package (Yu et al., 2012), where significant pathways with adjusted *P* value less than 0.05 was shown in Figure 4B. Besides, network topology-based analysis was performed on all DEGs based on the background network of TCGA sarcomas on the WebGestalt website (Zhang et al., 2005), where the top 10 neighbors were selected based on the probability of random walk method. All seeds and top-ranking neighbors in the expanded sub-network can enrich to 10 gene ontology (GO) biological process (BP) categories, as shown in Figure 4C. Also, the DEGs are only identified from the perspective of a single gene. However, due to the complexity of BPs, the analysis from the perspective of a single gene ignores the correlation of genes and cannot explore differences among TME groups from an overall perspective. Therefore, signaling pathway crosstalk analysis is performed on three TME groups from the level of the pathway, as shown in Figures 4D–F.

The heatmap of Figure 4A clearly shows the expression profiles of DEGs in three TME groups. It can be seen that most DEGs are downregulated genes, and the expression values are higher in the TME-C. In other words, when the gene expression value is higher, the survival rate is higher, but the group with lower expression value has a lower survival rate. In contrast, only a small number of DEGs expression values are lower, but the survival rate is also higher. In addition, after performing enrichment analysis on these DEGs, most of the KEGG pathways in Figure 4B are immune-related pathways, including T-cell-related pathways, immune deficiency-related pathways, PD-L1 expression, and PD-1 checkpoint pathway in cancer. The value of fold-change also indicates that the genes enriched in these pathways are all downregulated, and the GO enrichment analysis in Figure 4C shows that the pathways enriched by these DEGs are immune-related BPs. The shreds of evidence illustrated that most of DEGs in different sarcoma TME groups are immune-related genes, and most of the immune-related DEGs are highly expressed in the better prognostic group, but the expression level is lower in the worse prognostic group.

Moreover, signaling pathway crosstalk analysis shows that the pathway crosstalk patterns are different between TME groups. For TME-A and TME-B, the correlation between the pathways in TME-A is lower than that in TME-B. The main nodes are endocrine and other factor-regulated calcium reabsorption and dilated cardiomyopathy. There are also several crosstalk changes between multiple cancer-related pathways and immune cell pathways. A comparison of the pathway crosstalk between TME-A and TME-C shows that there is a higher correlation between multiple cancer pathways and Huntington’s disease pathway in TME-A. However, the correlation between antigen processing and presentation pathway and other disease-related pathways is lower in TME-C. Compared with TME-C, the higher correlations between the pathways occur in TME-B, where the largest node is the dorso-ventral axis formation pathway. The correlation between the dorso-ventral axis formation pathway and immune-related pathways is higher in TME-B. Therefore, although there are several overlaps of the significant signaling pathways in pairs of TME groups, most of them are not the same. What’s more, crosstalk analysis between each pair of TME groups reveals that information flow between the significant signaling pathways also begins to occur great changes when the TME changes. The crosstalk pathways in three comparison groups contain different disease pathways and immune pathways. Both of Fc gamma R-mediated phagocytosis and natural killer cell mediated cytotoxicity pathways are included between TMEA and TMEB, and between TME-B and TME-C. The difference is that more B cell-related crosstalk pathways appear in TME-A versus to TME-C. Also, more T-cell-related crosstalk pathways appear in TME-B versus to TME-C.

As mentioned earlier, the survival rate of patients in TME-C is higher, the survival rate of patients in TME-A is middle, and the survival rate of patients in TME-B is the lowest. Therefore, during the process of decreasing survival rate, the Antigen processing, and presentation pathway and T cell pathways are more active. B-cell-related pathways are more active in TME-A compared to TME-B. In the large span from TME-C to TME-B, the T-cell-related pathways and the natural killer cell pathway have been pretty active. It is known to all that regulatory T cells help control the immune response and prevent it from getting out of control. Natural killer T cells also produce chemicals to help regulate the immune response and prevent invaders and tumors. Therefore, our study also suggests that this phenomenon is also the same in different TME situations. The activity of immune cells changes as the microenvironment changes. The crosstalk between significant signaling pathways also changes following a specific law.

### Analysis of DNA Methylation Between Different TME Groups

**FIGURE 5:**
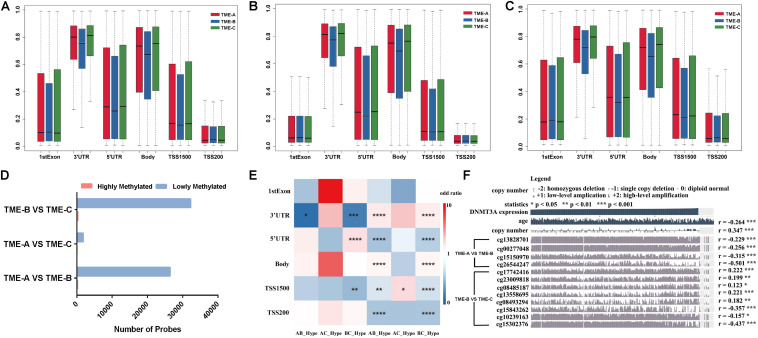
DNA methylation differences among the three TME groups. **(A)** Probe distribution of mean β value across differentially DNA methylated regions classified by gene annotations. The vertical axis represents the mean methylation value. **(B)** The mean β values of probes annotated to upregulated genes in the TME-B group versus the TME-C group across six genomic regions. **(C)** The mean β values of probes annotated to downregulated genes in the TME-B group versus the TME-C group across six genomic regions. **(D)** The number of hypermethylated and hypomethylated probes among TME groups. **(E)** The performance of hypermethylated and hypomethylated probes on genomic region enrichment based on six pairs of TME groups. The number of hypermethylated, hypomethylated, and total probes are calculated, and the odds ratio is calculated by Fisher’s test and Chi-square test shown by heatmap. The *P* value is labeled with asterisks in each grid (****P < 0.0001, ***P < 0.001, **P < 0.01, *P < 0.05, and ns: no significance). The horizontal axis indicates that hypomethylated probes or hypomethylated probes in TME groups (AB represents that TME-A versus TME-B; AC represents that TME-A versus TME-C; BC represents that TME-B versus TME-C; Hype represents hypermethylated probes; Hypo represents hypomethylated probes). **(F)** Correlation between DNMT3A expression and age, as well as between copy number and DNA methylation CpG sites. The age is at initial pathologic diagnosis.

In order to identify whether there is an absolute difference in DNA methylation between TME subtypes, DNA methylation was divided into six types according to the gene annotation. Figure 5 shows the distribution performance of gene annotation regions in three TME groups, with particular attention paid to differential DNA methylation between TME-B with worse prognosis and TME-C with better prognosis, including differential methylation positions corresponding to annotated upregulated genes or downregulated genes. Figure 5A describes the probe distribution of mean β value across differentially DNA methylated regions classified by gene annotations. Figure 5B shows the mean β values of probes annotated to upregulated genes in the TME-B group versus the TME-C group across six genomic regions. Figure 5C describes the mean β values of probes annotated to downregulated genes in the TME-B group versus the TME-C group across six genomic regions. At the same time, the differential analysis between two TME groups was used to divide the methylation sites into hypermethylation and hypomethylation groups and to count the number distribution, as shown in Figure 5D. The enrichment of the two types of differential methylation data in microenvironment group pairs has been shown in Figure 5E. The correlation between the expression of methyltransferases DNMT3A and the beta value of differential DNA methylation sites was also analyzed by using MEXPRESS (Koch et al., 2015), as shown in Figure 5F.

Compared with the other TME groups, TME-B shows lower methylation levels in most of the gene annotation regions, especially 3’utr, 5’utr, and body regions, as shown in Figure 5A. This trend of the corresponding differential methylation data of annotated genes has not been changed in Figures 5B,C, which indicates that the gene expression changes will affect the methylation of these three regions, no matter the expression of genes is upregulated or downregulated. For TSS1500, TME-B reveals a significantly lower level of methylation, especially when the annotated gene is downregulated. This evidence indicates that the decrease of gene expression levels may cause a significant decrease in the methylation level of the gene TSS1500 region. Moreover, the methylation levels in the 1stExon and TSS200 regions are not affected by changes in the gene expression levels. The distribution of hypermethylated and hypomethylated probes suggested that the proportion of hypermethylated in all three TME groups is higher, especially between TME-B and TME-C or TME-A and TME-B, as shown in Figure 5D. This result is also consistent with that the overall methylation level of TME-B is lower shown in the previous finding, which is the highest methylation level in TME-C, the intermediate methylation level in TME-A and the lowest methylation level in TME-B.

Figure 5E also shows some impressive results. From the perspective of regional classification, there is no significant difference in the 1stExon region regardless of the group, and there is no significant difference for hypermethylation in the body and TSS200 regions. However, there are significant differences in the distribution of hypomethylation in the TSS1500 region among three TME groups. Between TME-A and TME-C, there are almost no differences in the methylation sites, and only the hypomethylated probes are significantly different in the TSS1500 region. Also, the TME-B group and the other two groups have significant differences in hypermethylation and hypomethylation in the 3’UTR region. The difference in hypomethylation is noticeable, especially in other regions except 1stExon. These results are consistent with the previous results that the changes in gene expression level correlate strongly with the low methylation level of most gene annotation regions.

DNA methyltransferase is an enzyme responsible for transferring methyl to DNA. Gene epigenetics caused by defects in DNA methylation transferase are often accompanied by tumor occurrence and development. In this study, multiple significant DNA methylation sites correspond to the same methyltransferase DNMT3A in the comparison of TME-B with the other TME groups. The expression of DNMT3A shows a significant negative correlation with patient’s age and a significant positive correlation with the expansion and deletion of the copy number. Besides, it is noted that there are more significant differences between TME-B and TME-C, the beta value of which is related to the expression level of DNMT3A. The beta value of all significant differential DNA methylation sites between TME-A and TME-B is negatively related to the expression of DNMT3A. In addition, DNMT3A regulates the expression level of the gene by controlling the methylation status of the gene’s promoter region, which is also in line with the significant differences in TSS200 and TSS1500 belong to promoter regions between TME-B and other TME groups. Therefore, DNMT3A in different TMEs causes the methylation status of gene promoter regions to be significantly different, which affects gene expression.

### Analysis of CNV Between Different TME Groups

**FIGURE 6:**
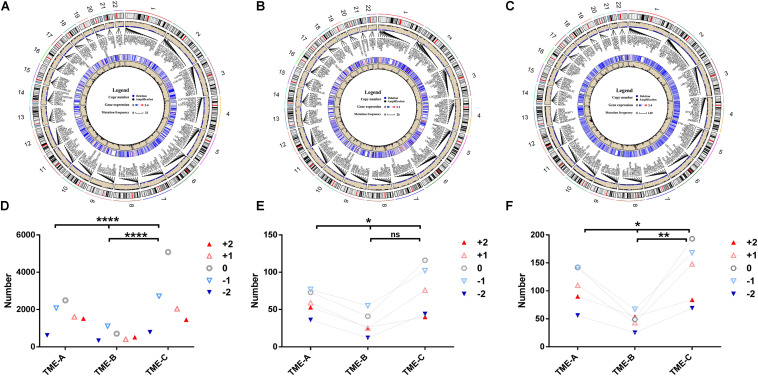
Copy number differences among the three TME groups. **(A–C)** The circular visualization of copy number performance across 22 autosomes in TME-A **(A)**, TME-B **(B)**, and TME-C **(C)**. The outermost ring represents the human chromosome data. The scatter plot represents the CNV labeled with several annotated representative genes symbols. Blue dots denote deletion, black dots denote amplification, and null denotes that there is no CNV. The heatmap represents the mean expression levels of annotated genes. The line plot represents the frequency of annotated genes. **(D)** The mutation number of different CNVs among three TME groups. **(E)** The mutation number of different CNVs annotated to genes that are upregulated in the TME-B versus TME-C. **(F)** The mutation number of different CNVs annotated to genes that are downregulated in the TME-B versus TME-C (****P < 0.0001, ***P < 0.001, **P < 0.01, *P < 0.05, ns: no significance; +2: homozygous deletion, -1: single copy deletion, 0: diploid normal, +1: low-level amplification, +2: high-level amplification).

As the same as DNA methylation, CNV exerts an enormous function on affecting gene expression. The mutation of the CNV site is also one of the important pathogenic factors of human diseases. In order to explore the discrepancies in CNV under TME groups, the *RCircos* R package (Zhang et al., 2013) was applied to plot the CNV on 22 chromosomes in three microenvironment states, as shown in Figures 6A–C. At the same time, the Chi-square test was used to calculate the significant difference in the distribution of CNV in different TMEs, as shown in Figures 6D–F.

There were more variations on chromosome 1 and chromosome 12 than those on other chromosomes in three TMEs. Compared with the other two groups, the frequency of copy number amplification of TME-B is significantly higher than the frequency of copy number deletion (deletion: amplification = 1: 1.8). In contrast, the frequency of amplification and deletion is similar in the other two cases (TME-A: 1: 1.1, TME-B: 1: 1.3). For the genes annotated by CNV, the gene expression data of TME-C was lower than that of the other two groups through the heatmaps. The line plot revealed that the mutation frequency of the genes was significantly higher than that of the other two groups. It indicates that the low-expressed genes may be more prone to CNV in the microenvironment with a better prognosis.

According to the results of Chi-square test, the distribution of the number of different CNVs is also significantly different in the three groups of microenvironments, which is also consistent with those above that the frequency of copy number deletion in TME-B is significantly higher than the frequency of copy number amplification. Considering that Dedifferentiated liposarcoma is heavily controlled by CNV, we have studied the CNV performance of Dedifferentiated liposarcoma, as shown in the Supplementary Figure S1. The results revealed the mutation performance of Dedifferentiated liposarcoma is different from the overall mutation performance. For Dedifferentiated liposarcoma, the frequency of copy number amplification in each TME groups is significantly higher than the frequency of copy number deletion, which is consistent with the previous studies on mutational profiles and genomic alterations in the Dedifferentiated liposarcoma (The Cancer Genome Atlas Research Network,, 2017). Furthermore, there is no significant difference between each pair of TME groups, although there are significant differences between the three groups in the CNV associated with the upregulated genes. On the other hand, compared with TME-C, the number of high-level amplifications is greater than the number of low-level amplifications in TME-B, which indicates that the CNV of highly expressed genes is more likely to occur at high levels of amplification in the TME with a worse prognosis.

### Analysis of ceRNA Networks Between Different TMEs

**FIGURE 7:**
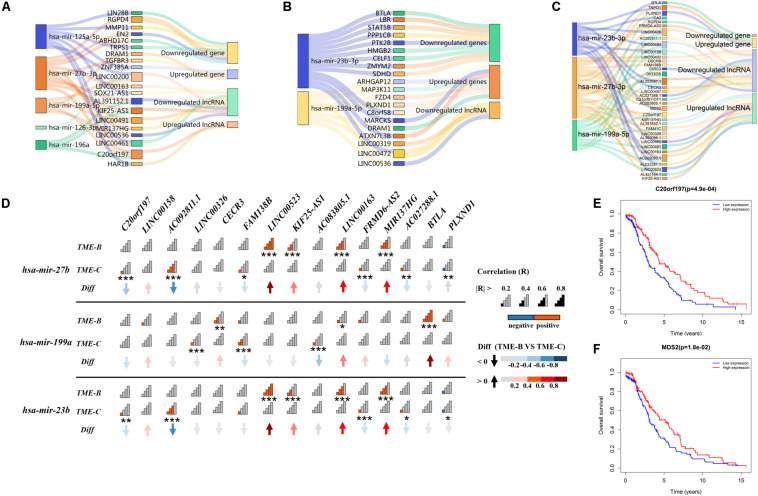
The Sankey plot of the endogenous competitive network in three TME groups. The rectangle size of miRNA represents the number of DEGs and lncRNAs. Panel **(A)** represents the ceRNA network of differentially expressed miRNA, lncRNA, and genes in the TME-A versus TME-B. Panel **(B)** represents the ceRNA network of differentially expressed miRNA, lncRNA, and genes in the TME-A versus TME-C. Panel **(C)** represents the ceRNA network of differentially expressed miRNA, lncRNA, and genes in the TME-B versus TME-C. Panel **(D)** represents the correlation of differentially expressed miRNA and lncRNAs between TME-B and TME-C. The “Diff” is defined as the differential correlation in TME-B versus TME-C. Signal plot shows the degree of correlation. The *P* value is labeled with asterisks below signal plot (***P < 0.001, **P < 0.01, *P < 0.05). **(E,F)** The Kaplan–Meier survival analysis for differentially expressed lncRNAs in TME-B versus TME-C. Red (blue) line represents the survival probability of patients with sarcomas when the expression level of RNAs is high (low).

Long non-coding RNAs can regulate the interaction between tumor cells and microenvironment and then affect tumorigenesis, development, and metastasis. Besides, lncRNA with the same miRNA response element can also bind to miRNA, so that the mRNA will compete with lncRNA to a certain degree, which indirectly regulates the expression level of mRNA and thus regulates cell function. In order to explore the competition mechanism between RNAs in three TMEs, this study uses DEGs, lncRNA, and miRNA between each pair of TME groups to build an endogenous competitive network as shown in Figure 7 to observe those differentially expressed molecules and the mechanism of how molecules competitively combine miRNAs, which in turn affects tumor development in the microenvironment.

As a whole, there are several overlapped competitive miRNAs among three TME groups, mainly including has-mir-199a, has-mir-23b, and has-mir-27b. It can be seen that mir-27b-3p regulates 5 same lncRNAs in TME-A versus to TME-B and TME-B versus to TME-C. Compared with others, the expression level of mir-27b-3p and C20orf197 are lower, but the expression level of KIF25-AS1, MIR137HG, LINC00163, and LINC00491 are higher in TME-B. However, when the microenvironment changes from TME-B to TME-C, the affected genes in the ceRNA network are not the same, but the same lncRNA will affect the same gene through different miRNAs. For example, TNPO1 will be regulated by mir-23b-3p and mir-27b-3p, as shown in Figure 7C. When AC009093.1, MDS2, and LINC00426 compete with two miRNAs, the expression value of miRNAs will decrease, which will inhibit the negative regulation of TNPO1, resulting in increased expression of TNPO1. This is also consistent with that lncRNA can indirectly inhibit the negative regulation of target genes by miRNAs by competitively binding with the 3’UTR of the target gene mRNA. The expression levels of mir-27b and mir-23b are higher in TME-C compared with TME-B. From the correlation diagram of miRNA and lncRNA, mir-27b and mir-23b show the same trend, which is consistent with that both belong to the same family with only one different nucleotide that has the effect of inhibiting tumor development. Compared with mir-27b/23b, the expression level of mir-199a is lower in TME-C, and the performance on correlation with lncRNA is also significantly different from the other two miRNAs. It indicates that different miRNAs have the same effect of inhibiting tumor growth, but the expression of miRNA in different TMEs also has individual differences, which regulated the expression value of downstream molecular.

In particular, the survival curve analysis of differentially expressed lncRNAs is performed using the “survival” R package. This univariate survival analysis was evaluated based on Kaplan–Meier curve analysis, and a *P* value of less than 0.05 was considered significant. The expression values of the two lncRNAs have a significant correlation with the survival time of patients. When the expression values of lncRNA C20orf197 and MDS2 are higher, the overall survival rate of patients is higher, consistent with the low expression of lncRNA in the worst prognostic group. Also, C20orf197 and MDS2 are at the hub of the ceRNA network, linking all miRNAs and multiple competing lncRNAs and genes. Therefore, two RNA molecules with a potential prognostic value play a role key role when the microenvironment changes significantly.

## Discussion and Conclusion

In this study, it is the first time to use gene expression data of over 800 sarcomas samples to identify the subtypes of TMEs and to analyze the difference from multiple dimensional molecular data. The infiltration degree of 22 kinds of immune cells in each sample was estimated, which was applied to divide all samples into three TME groups. Furthermore, the difference between five aspects, including DEGs, pathway crosstalk, DNA methylation, CNV, and endogenous completive network were identified.

Interactions between tumor cells and TME can help determine tumor progression. The distribution of immune cells in the TME determines the fate of tumor cells, which influences the survival of sarcoma patients. The present study divided these patients into three TME groups. The results revealed significant differences in the survival of sarcoma patients of different TME groups of sarcomas. Moreover, different B cells and T cells do not show strong consistency, but there is a particular bias in different types of TMEs. The relative abundances of T cells CD4 memory resting and B cell naïve are higher in the better prognostic group, which is consistent with the function of T cells CD4 memory resting that are likely to fulfill a vital facilitator role in the maintenance and control of protective immune responses (Stockinger et al., 2006). Also, the B cell naïve belongs to mature B cells, the increase of which promotes the secretion of more plasma cells to fight the antigen. Petitprez et al. have confirmed that B cells are the strongest prognostic factor, even in the context of high or low CD8+ T cells and cytotoxic contents (Petitprez et al., 2020). There is no significance for other T cells and B cells in the better prognostic group versus the worse prognostic group. Besides, the Macrophages M0 is significantly more active in the worst prognostic group, and the Macrophages M2 is significantly more active in the intermediate prognostic group. The Macrophages are the first line of defense against external infections, but recent studies have proved that tumor-associated macrophages accelerated tumor development, metastasis, and relapse (Hughes et al., 2015; Chen et al., 2017; Samaniego et al., 2018). The pieces of evidence are consistent with the relative higher abundances of macrophages cells.

The gene expression levels determine the relative immune cell abundances of each sample. It can be found that most of the common DEGs among three TME groups of sarcomas are immune-related genes, the gene expression levels of which change significantly in different TME groups. Most genes have higher expression values in the TME-C group with a better prognosis. The prognostic effect worsens, the expression levels of these genes also begin to show a downward trend. What’s more, the KEGG enrichment analysis revealed the relationship between these genes and enriched pathways. It is evident that these pathways are all immune-related, and all genes enriched in these pathways are downregulated genes in the worse prognostic group versus the better prognostic group. The enriched GO terms of DEGs and their top-ranking neighbors are evolved from the ancestor “immune system process.” These results are consistent with the immune cell inference results, as most of the immune cell infiltration is higher in the TME-C group with a better prognosis than that in the TME-B group with a worse prognosis. In addition, over 78% of downregulated genes are enriched the autoimmune diseases. For example, CD19 is widely present in B lymphocytes, and it has also been widely used in CART targeted therapy of B cell lymphoma (Ying et al., 2019).

Immune genes exert an enormous function in immune-related pathways and BPs among TMEs of sarcomas. However, these immune-related genes do not work in pathological processes alone but are regulated by upstream genes, which in turn affect the function of downstream genes (Deng et al., 2018a). Therefore, our study analyzes the level of crosstalk between signaling pathways in different microenvironments from the perspective of pathway levels to overcome the limitations of merely obtaining DEGs and analyzing them. According to the signaling pathway impact analysis, more cancer-related or tumor-related pathways were extracted, besides those immune-related pathways consistent with the results of the over-representing analysis. The pathways crosstalk relationships between the better prognostic group and intermediate prognostic group focus on the pathway of antigen processing and presentation, which is related to several signaling pathways. The information exchange between antigen processing and presentation pathway and other signaling pathways becomes more active in the worse prognostic TME group. Also, the crosstalk relationship between endocrine and other factor-regulated calcium reabsorption pathway and immune-related pathways or cancer-related pathways is more variable. These crosstalk relationships become weaker in the worst prognostic microenvironment. This evidence reveals that the differences in the TME will cause significant changes in the flow of information between pathways, especially when the degree of relative infiltration of immune cells is low, and the patient’s endocrine system may also be greatly affected, such as the absorption of trace molecules.

Mutation information exists in every region of the human chromosome, and these mutations regulate gene expression. The present study analyzes the performance of genetic variation information among three TME groups of sarcomas. DNA methylation sites and CNVs are significantly different in the three groups, and the common point is that the differentially expressed downregulated genes are more affected in the worse prognostic group versus better prognostic group. Separately, the worse prognostic group displayed the lowest DNA methylation level, especially in 3’UTR, 5’UTR, and body regions annotated to DEGs. Moreover, the expression level of DNA methyltransferase genes, DNMT3A, correlated negatively with age at initial pathologic diagnosis and correlated positively with copy number. Also, DNMT3A was related to many significantly differential DNA methylation CpG sites in different TMEs. A previous study has found that there is blastic plasmacytoid dendritic cell neoplasm with an unusual morphology, MYC rearrangement, TET2, and DNMT3A mutations (Kurt et al., 2018). Thus, different TMEs may cause the methylation status of gene promoter regions to be significantly different, which in turn affects gene expression. In addition, the CNV shows more amplification than deletion in the worse prognostic group. Although there were significant differences in the distribution of CNV among all DEGs, only the CNV corresponding to the downregulated genes was significantly different in the better prognostic group versus worse prognostic group. These results revealed that mutations in genetic information are more likely to have lower levels of methylation and fewer CNVs in the TME group with a worse prognosis than other TME groups.

Significant differences in different TMEs were found from both gene expression data and genetic variation data. Furthermore, the gene expression value is not only regulated by DNA methylation and CNV but also influenced by other molecules, such as miRNA and lncRNA. According to the endogenous competitive network constructed in three TME groups, it can be found that mir-27b differs between different TMEs and regulates different genes and lncRNAs. Previous studies have identified that miR-27b targets several genes to inhibit growth, tumor progression, and inflammatory response (Lee et al., 2012; Jin et al., 2013). The expression of mir-23b and mir-199a were also differentially expressed in osteosarcoma and regulated several signaling pathways to participate in various BPs of tumor cells (Duan et al., 2011; Liu et al., 2018). Notably, the correlation between miRNAs and lncRNAs was calculated and compared in a better prognostic group and worse prognostic group. Since mir-23b and mir-27b belong to the same family, the expression levels of the two miRNAs also showed a very high positive correlation. Several lncRNAs have been identified as the signatures of prognosis or tumor recurrence, such as AC092811.1, LINC00326, and C20orf197 (Yin et al., 2018; Zhu et al., 2019; Zhao et al., 2020). The correlation between miRNA and lncRNA is weaker in the TME with a worse prognosis than the TME with a better prognosis. It may be caused by the reason that the lncRNA becomes less competitive with the lncRNA expression level decreases, which causes miRNA to regulate more mRNA in turn.

Unfortunately, we performed Fisher’s test on the drug response of the different TME groups of patients and found no significant differences. The reason is owing to the sample size of the drug information used in this study is not enough so that the experimental results are too limiting. Therefore, in future experiments, this study will expand the sample and explore the situation in different TMEs from the level of immunotherapy and drug response.

In summary, a reasonable microenvironment classification mechanism was applied for a large amount of sarcoma data to divide samples into three TME phenotypes according to the relative infiltration of various immune cells. According to integrate multiple dimensional data, the present study revealed that there were many differences of multi-molecular levels among three TME groups related to prognosis. These findings can enhance our understanding of the prognostic factors in the TME of sarcomas. Furthermore, these prognostic molecules identified in this study have potential value in biomarker development and personalized medicine.

## Data Availability Statement

All datasets presented in this study are included in the article/Supplementary Material.

## Author Contributions

JD and WZ conceived and designed the study. JD performed the experiments. JD, WK, and YS collected the samples and analyzed the data. JD and WK wrote the manuscript. WZ and XM reviewed and edited the manuscript. All authors read and approved the final version of the manuscript.

## Conflict of Interest

The authors declare that the research was conducted in the absence of any commercial or financial relationships that could be construed as a potential conflict of interest.

## Footnotes

1 https://portal.gdc.cancer.gov/

2 https://genome.ucsc.edu/index.html

## References

[B1] Berghuis D. Santos S. J. Baelde H. J. Taminiau A. H. Egeler R. M. Schilham M. W. (2016). Pro-inflammatory chemokine-chemokine receptor interactions within the Ewing sarcoma microenvironment determine CD8 + T-lymphocyte infiltration and affect tumour progression. *J. Pathol.* 223 347–357. 10.1002/path.2819 21171080

[B2] Chen X. W. Yu T. J. Zhang J. Li Y. Chen H. L. Yang G. F. (2017). CYP4A in tumor-associated macrophages promotes pre-metastatic niche formation and metastasis. *Oncogene* 36 5045–5057. 10.1038/onc.2017.118 28481877 PMC5582214

[B3] Chou C. H. Chang N. W. Shrestha S. Hsu S. D. Lin Y. L. Lee W. H. (2016). miRTarBase 2016: updates to the experimentally validated miRNA-target interactions database. *Nucleic Acids Res.* 44 D239–D247. 10.1093/nar/gkv1258 26590260 PMC4702890

[B4] Daw N. C. Chou A. J. Jaffe N. Rao B. N. Billups C. A. Rodriguez-Galindo C. (2015). Recurrent osteosarcoma with a single pulmonary metastasis: a multi-institutional review. *Br. J. Cancer* 112 278–282. 10.1038/bjc.2014.585 25422914 PMC4453448

[B5] Delespaul L. Lesluyes T. Pérot G. Brulard C. Lartigue L. Baud J. (2017). Recurrent TRIO fusion in nontranslocation-related sarcomas. *Clin. Cancer Res.* 23 857–867. 10.1158/1078-0432.CCR-16-0290 27528700

[B6] Deng J. Kong W. Mou X. Y. Wang S. Q. (2018a). Pathway crosstalk analysis based on signaling pathway impact analysis in Alzheimer’s disease. *Curr. Proteom.* 15 142–150. 10.2174/1570164614666171030162949

[B7] Deng J. Kong W. Wang S. Q. Mou X. Y. Zeng W. M. (2018b). Prior knowledge driven joint NMF algorithm for ceRNA co-module identification. *Int. J. Biol. Sci.* 14 1822–1833. 10.7150/ijbs.27555 30443186 PMC6231218

[B8] Deng J. Zeng W. M. Kong W. Shi Y. H. Mou X. Y. Guo J. (2020a). Multi-constrained joint non-negative matrix factorization with application to imaging genomic study of lung metastasis in soft tissue sarcomas. *IEEE Trans. Bio Med. Eng.* 67:2211. 10.1109/TBME.2019.2954989 31751222

[B9] Deng J. Zeng W. M. Shi Y. H. Kong W. Guo S. J. (2020b). Fusion of FDG-PET image and clinical features for prediction of lung metastasis in soft tissue sarcomas. *Comput. Math. Method M.* 2020:8153295. 10.1155/2020/8153295 32454885 PMC7222598

[B10] Duan Z. Choy E. Harmon D. Liu X. Susa M. Mankin H. (2011). MicroRNA-199a-3p is downregulated in human osteosarcoma and regulates cell proliferation and migration. *Mol. Cancer Ther.* 10 1337–1345. 10.1158/1535-7163.MCT-11-0096 21666078 PMC3711153

[B11] Farzana A. Haass N. K. (2018). Microenvironment-driven dynamic heterogeneity and phenotypic plasticity as a mechanism of melanoma therapy resistance. *Front Oncol.* 8:173. 10.3389/fonc.2018.00173 29881716 PMC5976798

[B12] Garcia D. M. Baek D. Shin C. Bell G. W. Grimson A. Bartel D. P. (2011). Weak seed-pairing stability and high target-site abundance decrease the proficiency of lsy-6 and other microRNAs. *Nat. Struct. Mol. Biol.* 18 1139–1146. 10.1038/nsmb.2115 21909094 PMC3190056

[B13] Giuliano K. Sachs T. Montgomery E. Guzzetta A. Brock M. Pawlik T. M. (2016). Survival following lung metastasectomy in soft tissue sarcomas. *Thorac. Cardiov. Surg.* 64 150–158. 10.1055/s-0035-1563538 26339728

[B14] Grzywa T. M. Paskal W. Wlodarski P. K. (2017). Intratumor and intertumor heterogeneity in melanoma. *Transl. Oncol.* 10 956–975. 10.1016/j.tranon.2017.09.007 29078205 PMC5671412

[B15] Huang R. Z. Meng T. Chen R. Yan P. Zhang J. Hu P. (2019). The construction and analysis of tumor-infiltrating immune cell and ceRNA network in recurrent soft tissue sarcoma. *Aging* 11 10116–10143. 10.18632/aging.102424 31739284 PMC6914407

[B16] Hughes R. Qian B. Z. Rowan C. Muthana M. Keklikoglou I. Olson O. C. (2015). Perivascular M2 macrophages stimulate tumor relapse after chemotherapy. *Cancer Res.* 75 3479–3491. 10.1158/0008-5472.CAN-14-3587 26269531 PMC5024531

[B17] Jeggari A. Marks D. S. Larsson E. (2012). miRcode: a map of putative microRNA target sites in the long non-coding transcriptome. *Bioinformatics* 28 2062–2063. 10.1093/bioinformatics/bts344 22718787 PMC3400968

[B18] Jin L. Wessely O. Marcusson E. G. Ivan C. Calin G. A. Alahari S. K. (2013). Prooncogenic factors miR-23b and miR-27b are regulated by Her2/Neu, EGF, and TNF-A in breast cancer. *Cancer Res.* 73 2884–2896. 10.1158/0008-5472.CAN-12-2162 23338610 PMC3855090

[B19] Kalluri R. (2016). The biology and function of fibroblasts in cancer. *Nat. Rev. Cancer* 16 582–598. 10.1038/nrc.2016.73 27550820

[B20] Khatri P. Sirota M. Butte A. J. (2012). Ten years of pathway analysis: current approaches and outstanding challenges. *PLoS Comput. Biol.* 8:e1002375. 10.1371/journal.pcbi.1002375 22383865 PMC3285573

[B21] Koch A. Meyer T. D. Jeschke J. Criekinge W. V. (2015). MEXPRESS: visualizing expression, DNA methylation and clinical TCGA data. *BMC Genomics* 16:636. 10.1186/s12864-015-1847-z 26306699 PMC4549898

[B22] Kong W. Zhang J. M. Mou X. Y. Yang Y. (2014). Integrating gene expression and protein interaction data for signaling pathway prediction of Alzheimer’s disease. *Comput. Math. Method* 2014:340758. 10.1155/2014/340758 24812571 PMC4000644

[B23] Kurt H. Khoury J. D. Medeiros L. J. Huh Y. (2018). Blastic plasmacytoid dendritic cell neoplasm with unusual morphology, MYC rearrangement and TET2 and DNMT3A mutations. *Br. J. Haematol.* 181:305. 10.1111/bjh.15128 29411884

[B24] Lee J. J. Drakaki A. Iliopoulos D. Struhl K. (2012). MiR-27b targets PPARγ to inhibit growth, tumor progression and the inflammatory response in neuroblastoma cells. *Oncogene* 31 3818–3825. 10.1038/onc.2011.543 22120719 PMC3290753

[B25] Leonardo S. Laura P. Yvonne T. Lev K. Pandolfi P. P. (2011). A ceRNA Hypothesis: the rosetta stone of a hidden RNA language? *Cell* 146 353–358. 10.1016/j.cell.2011.07.014 21802130 PMC3235919

[B26] Lesluyes T. Pérot G. Largeau M. R. Brulard C. Lagarde P. Dapremont V. (2016). RNA sequencing validation of the complexity INdex in SARComas prognostic signature. *Eur. J. Cancer* 57 104–111. 10.1016/j.ejca.2015.12.027 26916546

[B27] Li J. H. Liu S. Zhou H. Qu L. H. Yang J. H. (2014). starBase v2.0: decoding miRNA-ceRNA, miRNA-ncRNA and protein-RNA interaction networks from large-scale CLIP-Seq data. *Nucleic. Acids. Res.* 42, D92–D97. 10.1093/nar/gkt1248 24297251 PMC3964941

[B28] Li X. Cai Y. P. (2019). Better prognostic determination and feature characterization of cutaneous melanoma through integrative genomic analysis. *Aging* 11 5081–5107. 10.18632/aging.102099 31322504 PMC6746212

[B29] Liu H. Wei W. Wang X. Guan X. Chen Q. Pu Z. (2018). miR-23b-3p promotes the apoptosis and inhibits the proliferation and invasion of osteosarcoma cells by targeting SIX1. *Mol. Med. Rep.* 18 5683–5692. 10.3892/mmr.2018.9611 30387818

[B30] Liu K. W. Yang Y. (2018). Incorporating link information in feature selection for identifying tumor biomarkers by using miRNA-mRNA paired expression data. *Curr. Proteom.* 15 165–171. 10.2174/1570164614666171031160232

[B31] Mantovani A. Marchesi F. Malesci A. Laghi L. Allavena P. (2017). Tumour-associated macrophages as treatment targets in oncology. *Nat. Rev. Clin. Oncol.* 14 399–416. 10.1038/nrclinonc.2016.217 28117416 PMC5480600

[B32] Newman A. M. Liu C. L. Green M. R. Gentles A. J. Feng W. G. Xu Y. (2015). Robust enumeration of cell subsets from tissue expression profiles. *Nat. Methods* 12 453–457. 10.1038/nmeth.3337 25822800 PMC4739640

[B33] Paydas S. Bagir E. K. Deveci M. A. Gulfiliz G. (2016). Clinical and prognostic significance of PD-1 and PD-L1 expression in sarcomas. *Med. Oncol.* 33:93. 10.1007/s12032-016-0807-z 27421997

[B34] Petitprez F. Reyniès A. D. Keung E. Z. Chen T. W. Sun C. M. Calderaro J. (2020). B cells are associated with survival and immunotherapy response in sarcoma. *Nature* 577 556–560. 10.1038/s41586-019-1906-8 31942077

[B35] Quail D. F. Joyce J. A. (2013). Microenvironmental regulation of tumor progression and metastasis. *Nat. Med.* 19 1423–1437. 10.1038/nrc2618 24202395 PMC3954707

[B36] Ritchie M. E. Belinda P. Di W. Hu Y. F. Law C. W. Shi W. (2015). limma powers differential expression analyses for RNA-sequencing and microarray studies. *Nucleic Acids Res.* 43:e47. 10.1093/nar/gkv007 25605792 PMC4402510

[B37] Rooney M. S. Shukla S. A. Wu C. J. Getz G. Hacohen N. (2015). Molecular and genetic properties of tumors associated with local immune cytolytic activity. *Cell* 160 48–61. 10.1016/j.cell.2014.12.033 25594174 PMC4856474

[B38] Safonov A. Jiang T. Bianchini G. Gyõrffy B. Karn T. Hatzis C. (2017). Immune gene expression is associated with genomic aberrations in breast cancer. *Cancer Res.* 77 3317–3324. 10.1158/0008-5472.CAN-16-3478 28428277

[B39] Samaniego R. Alejandra G. G. Alba G. S. Jorge G. G. Enrique M. Iván M. R. (2018). CCL20 expression by tumor-associated macrophages predicts progression of human primary cutaneous melanoma. *Cancer Immunol. Res.* 6 267–275. 10.1158/2326-6066.CIR-17-0198 29362221

[B40] Stahl D. Gentles A. J. Thiele R. Guetgemann I. (2019). Prognostic profiling of the immune cell microenvironment in ewing’s sarcoma family of tumors. *Oncoimmunology* 2019:e1674113. 10.1080/2162402X.2019.1674113 31741777 PMC6844324

[B41] Steele C. D. Pillay N. (2019). The genomics of undifferentiated sarcoma of soft tissue: progress, challenges and opportunities. *Semin. Cancer Biol.* 61 42–55. 10.1016/j.semcancer.2019.11.009 31866474

[B42] Stefano M. Pablo T. Jill M. Todd G. (2003). Consensus clustering: a resampling-based method for class discovery and visualization of gene expression microarray data. *Mach. Learn.* 52 91–118. 10.1023/a:1023949509487

[B43] Stockinger B. Bourgeois C. Kassiotis G. (2006). CD4+ memory T cells: functional differentiation and homeostasis. *Immunol. Rev.* 211 39–48. 10.1240/sav_gbm_2009_m_002356 16824115

[B44] Tarca A. L. Draghici S. Khatri P. Hassan S. (2009). A novel signaling pathway impact analysis. *Bioinformatics* 25 75–82. 10.1093/bioinformatics/btn577 18990722 PMC2732297

[B45] The Cancer Genome Atlas Research Network Adam A. Clement A. Sally N. A. Rehan A. Teniola A. (2017). Comprehensive and integrated genomic characterization of adult soft tissue sarcomas. *Cell* 171 950–965. 10.1016/j.cell.2017.10.014 29100075 PMC5693358

[B46] Thorsson V. Gibbs D. L. Brown S. D. Wolf D. Bortone D. S. Yang T. H. (2018). The immune landscape of cancer. *Immunity* 48 812–830. 10.1016/j.immuni.2018.03.023 29628290 PMC5982584

[B47] Tirosh I. Izar B. Prakadan S. M. Wadsworth M. H. Treacy D. Trombetta J. J. (2016). Dissecting the multicellular ecosystem of metastatic melanoma by single-cell RNA-seq. *Science* 352 189–196. 27124452 10.1126/science.aad0501 PMC4944528

[B48] Wong N. Wang X. (2015). miRDB: an online resource for microRNA target prediction and functional annotations. *Nucleic Acids Res.* 43 D146–D152. 10.1093/nar/gku110 25378301 PMC4383922

[B49] Yang Y. Wu Z. C. Kong W. (2018). Improving clustering of microRNA microarray data by incorporating functional similarity. *Curr. Bioinform.* 13 34–41. 10.2174/1574893611666160711162634

[B50] Yang Y. Xiao Y. Q. Cao T. Y. Kong W. (2017). MiRFFS: a functional group-based feature selection method for the identification of microRNA biomarkers. *Int. J. Data Min. Bioin.* 18 40–55. 10.1504/IJDMB.2017.10007184

[B51] Yin X. Huang S. Zhu R. Fan F. Sun C. Hu Y. (2018). Identification of long non-coding RNA competing interactions and biological pathways associated with prognosis in pediatric and adolescent cytogenetically normal acute myeloid leukemia. *Cancer Cell Int.* 18:122. 10.1186/s12935-018-0621-0 30181715 PMC6114287

[B52] Ying Z. Huang X. F. Xiang X. Liu Y. Kang X. Song Y. (2019). A safe and potent anti-CD19 CAR T cell therapy. *Nat. Med.* 6 947–953. 10.1038/s41591-019-0421-7 31011207 PMC7518381

[B53] Yu G. C. Wang L. G. Han Y. Y. Han Q. Y. (2012). clusterProfiler: an R package for comparing biological themes among gene clusters. *OMICS* 16 284–287. 10.1089/omi.2011.0118 22455463 PMC3339379

[B54] Zeng D. Q. Li M. Y. Zhou R. Zhang J. W. Sun H. Y. Shi M. (2019). Tumor microenvironment characterization in gastric cancer identifies prognostic and immunotherapeutically relevant gene signatures. *Cancer Immunol. Res.* 7 737–750. 10.1158/2326-6066.CIR-18-0436 30842092

[B55] Zhang B. Kirov S. Snoddy J. (2005). WebGestalt: an integrated system for exploring gene sets in various biological contexts. *Nucleic Acids Res.* 33 W741–W748. 10.1093/nar/gki475 15980575 PMC1160236

[B56] Zhang H. Meltzer P. Davis S. (2013). RCircos: an R package for Circos 2D track plots. *BMC Bioinform.* 14:244. 10.1186/1471-2105-14-244 23937229 PMC3765848

[B57] Zhao D. F. Peng Q. Wang L. Li C. Lv Y. Liu Y. (2020). Identification of a six-lncRNA signature based on a competing endogenous RNA network for predicting the risk of tumour recurrence in bladder cancer patients. *J. Cancer* 11 108–120. 10.7150/jca.35801 31892978 PMC6930402

[B58] Zhu Y. Sun X. W. Lin J. Zhang T. Liu X. Shen X. (2019). Investigating potential molecular mechanisms of carcinogenesis and genes as biomarkers for prognosis of gastric cancer based on integrated bioinformatics analysis. *Pathol. Oncol. Res.* 25 1125–1133. 10.1007/s12253-018-0523-4 30430424 PMC6614141

