# Supplementary figures and images for: The Study of Sarcoma Microenvironment Heterogeneity Associated With Prognosis Based on an Immunogenomic Landscape Analysis
Source: Front Bioeng Biotechnol. 2020 Aug 21;8:1003. doi: 10.3389/fbioe.2020.01003 (PMC7471631; doi:10.3389/fbioe.2020.01003)

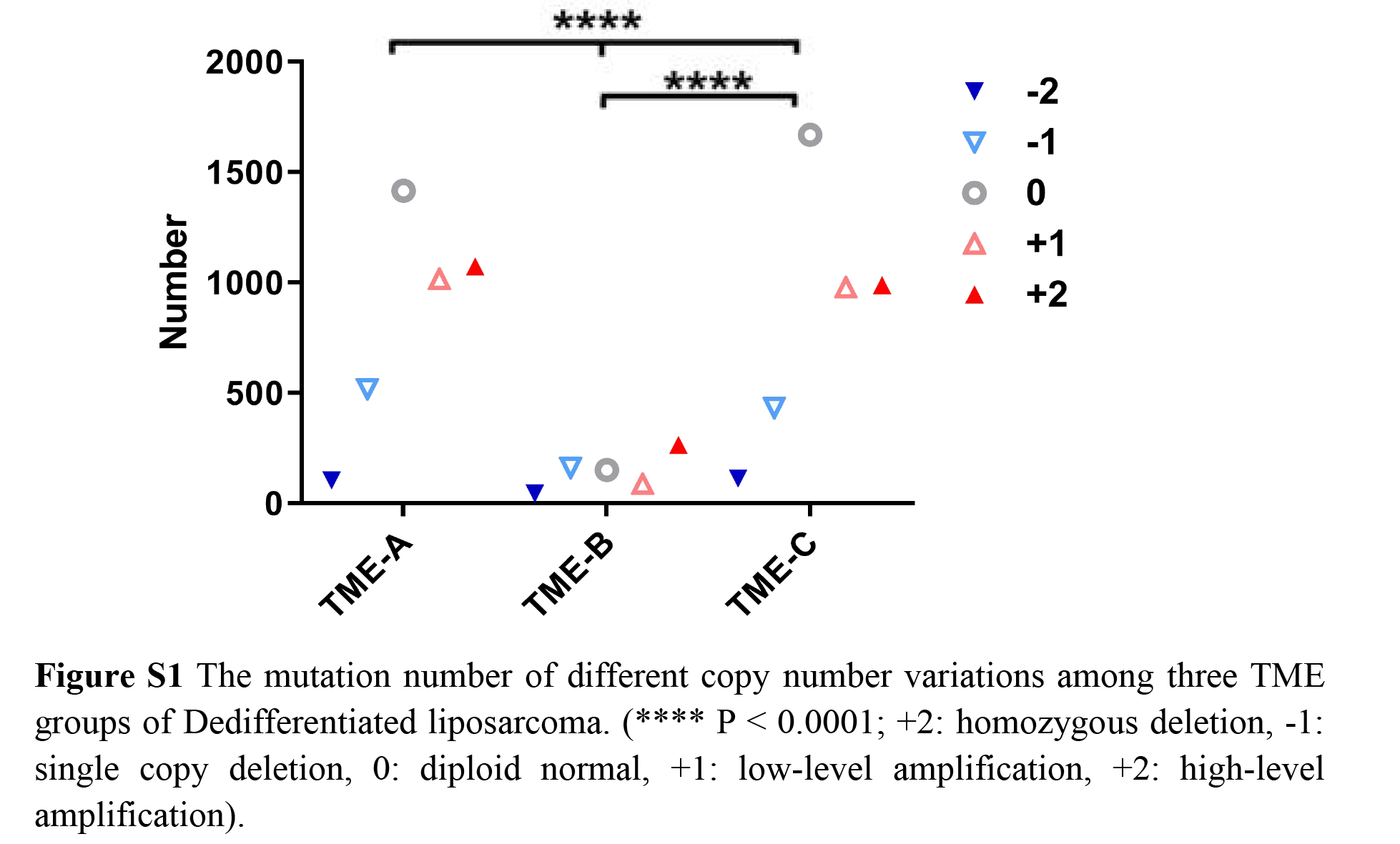

Supplement: Supplementary file 1 [file Image_1.TIF]
